# Heart Failure: Advanced Development in Genetics and Epigenetics

**DOI:** 10.1155/2015/352734

**Published:** 2015-04-09

**Authors:** Jian Yang, Wei-wei Xu, Shen-jiang Hu

**Affiliations:** Department of Cardiology, The First Affiliated Hospital, College of Medicine, Zhejiang University, No. 79, Qing-Chun Road, Hangzhou 310003, China

## Abstract

Heart failure (HF) is a complex pathophysiological syndrome that arises from a primary defect in the ability of the heart to take in and/or eject sufficient blood. Genetic mutations associated with familial dilated cardiomyopathy, hypertrophic cardiomyopathy, and arrhythmogenic right ventricular cardiomyopathy can contribute to the various pathologies of HF. Therefore, genetic screening could be an approach for guiding individualized therapies and surveillance. In addition, epigenetic regulation occurs via key mechanisms, including ATP-dependent chromatin remodeling, DNA methylation, histone modification, and RNA-based mechanisms. MicroRNA is also a hot spot in HF research. This review gives an overview of genetic mutations associated with cardiomyopathy and the roles of some epigenetic mechanisms in HF.

## 1. Background

Heart failure (HF) is a complex pathophysiological syndrome that arises from a primary defect in the ability of the heart to take in and/or eject sufficient blood [1]. The clinical manifestations of HF mainly arise from the primary myocardial disease, most commonly coronary artery disease, hypertension, and inherited cardiomyopathy. Although the etiology is highly variable, HF represents a derangement of the interplay between the cardiac, renal, and vascular systems. Without successful intervention, inadequate systolic or diastolic cardiac function causes poor systemic blood flow, leading to compensatory neurohormone release, vasoconstriction, and fluid retention. HF remains the most devastating cardiovascular disease in terms of morbidity, mortality, quality of life, and health care costs and is still the number one killer in the western world. It is estimated that symptomatic HF currently affects 0.4%–2% of the general population in the western world [2]. However, importantly, the incidence of symptomatic HF increases substantially with increasing age, and the prevalence of symptomatic HF in individuals over 65 years of age is estimated to be 6%–10%. Up to 50% of patients diagnosed with HF will die within four years, and for patients with end-stage HF, the one-year-survival rate is 50%, which is worse than most advanced malignancies [2].

## 2. Genetics

Genetic mutations can contribute to the various pathologies of HF by altering the structures and functions of the proteins responsible for various cellular activities. The current classification of cardiomyopathies by the European Society of Cardiology recognizes four major types of cardiomyopathies: hypertrophic cardiomyopathy (HCM), dilated cardiomyopathy (DCM), restrictive cardiomyopathy (RCM), and arrhythmogenic right ventricular cardiomyopathy (ARVC) as well as unclassified cardiomyopathies, such as noncompaction cardiomyopathy (NCCM) [3]. In the past few years, over 100 cardiomyopathy-related genes have been identified. Approximately 30 genes have been identified that are associated with HCM, 40 with DCM, 10 with RCM, five with ARVC, and 10 with NCCM [4–6]. Here, we present detailed introductions to familial dilated cardiomyopathy (FDCM), familial hypertrophic cardiomyopathy (FHCM), and ARVC.

### 2.1. Dilated Cardiomyopathy

Modern familial screening studies have shown that up to 48% of DCM may be inherited [7]. To date, mutations in more than 40 genes have been implicated in the development of DCM [8]. Inherited DCM is usually transmitted as an autosomal dominant trait, although all other single inheritance patterns have been identified (autosomal recessive, X-linked, and mitochondrial) [7]. In a recent study of 312 patients, FDCM with a phenotype of high age penetrance and worse outcomes in males, which occurred in 27%, was attributed to truncation mutations in titin (TTN) [9, 10]. Genome-wide mapping and exome sequencing in a unique affected family have identified GATAD1, encoding the GATA zinc finger domain containing protein 1, as another pathogenic gene causing autosomal recessive DCM [11, 12]. The main DCM-causing genes are shown in Table 1.

### 2.2. Hypertrophic Cardiomyopathy

HCM is characterized as left ventricular hypertrophy in the absence of abnormal loading conditions sufficient to explain the observed degree of hypertrophy [13]. Recent studies have shown that 67% of HCM is inherited [14]. HCM is caused by mutations in genes encoding myofilament proteins of the sarcomere, Z-disc proteins, Ca^2+^-handling proteins, and other proteins related to the sarcomere. The majority of patients with FHCM show autosomal dominant mutations in genes encoding sarcomere proteins, such as *β*-myosin heavy chain (*MYH7*), cardiac myosin-binding protein C (*MYBPC3*), cardiac troponin T (*TNNT2*), troponin I (*TNNI3*), alpha-tropomyosin (*TPM1*), myosin light chains (*MYL2* and* MYL3*), and cardiac actin (ACTC1) [15–17]. Mutations in genes encoding other sarcomere or related proteins, including alpha-myosin heavy chain (MYH6) and titin (TTN), genes encoding Z-disc proteins, such as muscle LIM protein (CSRP3), or genes encoding calcium-handling proteins (e.g., phospholamban) individually account for less than 1% of cases. Recently, the mitochondrial 16S rRNA 2336T>C mutation has been reported to be another pathogenic gene alteration associated with HCM, when the RNA of another unique family was sequenced [18].

The main HCM-related genes are shown in Table 2.

### 2.3. Arrhythmogenic Right Ventricular Cardiomyopathy

Arrhythmogenic right ventricular cardiomyopathy (ARVC) is increasingly designated arrhythmogenic cardiomyopathy because biventricular and left-dominant forms are recognized [19]. Around 40% of patients with ARVC carry pathogenic mutations in the similar genes as observed for other myocardium such as HCM and DCM [20]. In most patients, the disease is inherited as an autosomal dominant disease caused by mutations in one of the five genes encoding desmosomal proteins: plakophilin-2 (PKP2), desmoplakin (DSP), plakoglobin (JUP), desmoglein-2 (DSG2), and desmocollin-2 (DSC2). Nondesmosomal genes implicated in the disease include the transforming growth factor 3 (TGFB3), cardiac ryanodine receptor 2 (RYR2), and transmembrane protein 43 (TMEM43) [21]. A novel genetic variant in the transcription factor Islet-1 has been shown in recent studies to be associated with the onset of ARVC [22–24]. The main genes which are involved in ARVC are shown in Table 3.

Interestingly, the genes causing different types of cardiomyopathy overlap to a large extent, and mutations in the same gene may, in some instances, exert opposite functional effects [25, 26]. How the variations in a single gene generate different cardiomyopathic phenotypes is unknown, but current hypotheses under investigation involve transcriptional regulation, posttranslational modification, modifier variants of other genes, environmental influences, and the differential effects of the specific regions of the protein affected by the mutations. Detailed information is shown in Table 4.

### 2.4. Future Development: Gene Screening

Epidemiological studies that include family histories and echocardiographic screening of first-degree relatives have found that left ventricular remodeling and depressed fractional shortening are common in the asymptomatic relatives of patients with inherited cardiomyopathy and are associated with a statistically significant medium-term risk of disease progression [27–31]. Thus, genetic screening is extremely important because it can identify relatives at risk of developing the disease, allowing their early treatment and correct surveillance [28, 32, 33]. Family screening and genetic testing in families with a history of cardiomyopathy are recommended in clinical guidelines [34, 35]. In the last few years, next-generation sequencing techniques have improved the efficiency and speed of gene sequencing, and extensive and cheaper panels of DCM genes are now available, giving hope for the early detection of familial cardiomyopathy [36, 37].

Next-generation sequencing (NGS) technologies allow massively parallel DNA sequencing of gene panels, the whole coding sequence (exome) or the whole genome sequence (WGS) in a single experiment at an affordable price and a timeline of days or weeks, a significant advantage over traditional Sanger sequencing [38]. NGS technologies offer the advantage of unbiased genome-wide variant detection in small nuclear families and sporadic cases that previously could not be used effectively with traditional linkage analysis. Presently, the speed at which NGS can identify novel genetic variants of unknown significance is far greater than the speed at which functional assays can be used to assess variant pathogenicity [20].

However, the use of genetic testing in clinical practice is not common today, for reasons including the cost and complexity of the sequencing technologies. Moreover, the relatively low quality of genetic testing can detect pathogenic variants in only about 50% of individuals with familial HCM, DCM, or ARVC [8, 35, 39]. Another factor that limits the clinical application of genetics is our poor understanding of genotype-phenotype relationships [36]. Many of the clinical associations initially established for individual mutations have not been reproducible [40, 41]. An exception may be the poor prognoses for compound or double heterozygotes [42, 43], especially in individuals with HCM or ARVC. Patients carrying multiple mutations are more likely to present earlier and with more-severe disease. A genotype-phenotype association recently proposed in HCM showed increased cardiovascular events and the more frequent evolution to a dilated phenotype in the presence of any sarcomere-related gene mutation compared to genotype-negative patients [44, 45]. Finally, it is increasingly recognized that the genetic backgrounds of different inherited cardiomyopathies overlap substantially. This further challenges current attempts to establish genotype-phenotype relationships. Widely different phenotypes (dilation, noncompaction, and hypertrophy) can be caused by mutations in the same genes or even by the same mutations [36, 46].

In the future, continuing advances in the field will increase the uptake of genetic testing when lower costs make it a routine technology in daily clinical practice. As the number of patients who undergo genetic testing increases and the availability of genetic databases improves, the interpretation of genetic findings will become easier and more robust. The information obtained from large cohorts of patients carrying the same mutated genes will be the first step towards the design and adoption of genetically individualized therapies and surveillance.

## 3. Epigenetics

### 3.1. Chromatin Remodeling

There are four different families of ATP-dependent chromatin remodeling complexes: switching defective/sucrose nonfermenting complexes (SWI/SNF), imitation switch complexes, chromodomain-helicase-DNA-binding complexes, and inositol-requiring 80 complexes [47–49]. In this section, we will focus on the brahma-associated factor (BAF) complex, the vertebrate orthologue of the SWI/SNF complex, which was initially identified in* Saccharomyces cerevisiae*. In mammals, there are 14 BAF subunits, which contain either brahma (BRM) or brahma-related gene 1 (BRG1) as the ATPase subunit. Certain BAF subunits are only expressed in specific cell types, defining the tissue- or cell-type-specific BAF complexes. The BAF complex is involved in several cellular processes, including heart and muscle development [50]. BRG1 plays a key role in the switch from the fetal myosin heavy chain isoform (i.e., *β*-MHC or MYH7) to the adult MHC (*α*-MHC or MYH6) during cardiac hypertrophy. BRG1 is activated in HCM and its levels correlate with disease severity. Therefore, preventing the reexpression of BRG1 reduces hypertrophy and reverses the shift in myosin isoforms [51].

### 3.2. DNA Methylation

DNA methylation is the most common epigenetic modification in the mammalian genome [52, 53]. A genome-wide study of DNA methylation in the hearts of end-stage cardiomyopathic patients showed that methylation was significantly reduced in the promoters of upregulated genes but unchanged in the promoters of downregulated genes [54]. A recent genome-wide analysis of repetitive element methylation in the cardiac genome revealed that the hypomethylation of satellite elements was associated with significant upregulation of juxtacentromeric SATELLITE transcripts in diseased hearts compared with healthy controls [55]. Researchers also found an altered DNA methylation pattern in the myocardia of patients with idiopathic DCM, causing the misexpression of the genes for lymphocyte antigen 75 (LY75) and the tyrosine kinase-type cell surface receptor HER3 (ERBB3), the zebrafish orthologues of which are important for both adaptive and maladaptive responses in HF [56]. There is new evidence that increased DNA methylation plays a causative role in programming heart hypertrophy and reducing cardiac contractility, suggesting that demethylation is a potential therapeutic strategy in the treatment of HF and ischemic heart disease [57]. These studies support the possible role of DNA methylation in regulating the changes in gene expression that underlie HF. Movassagh et al. identified three angiogenic factors whose expression could be regulated by DNA methylation in human heart failure. Platelet/endothelial cell adhesion molecule 1 (PECAM1), angiomotin-like 2 (AMOTL2), and Rho GTPase activating protein 24 (ARHGAP24) showed possible novel genetic pathways through the process of methylation [54]. Expression of these methylating enzymes was shown to be regulated by hypoxia-inducible factor- (HIF-) 1alpha, which may prove to be a valuable therapeutic approach [58]. DNMT3A likely plays an essential role in RASSF1A mediated upregulation of ERK1/2 in rat cardiac fibrosis [59]. However, they do not clearly demonstrate whether these epigenetic markers are involved in regulating gene expression in cardiac hypertrophy or the stage in the pathology at which they are involved. Despite this, inhibitors of DNA methylation are a potential therapy for preventing cardiac hypertrophy and treating HF because they reverse norepinephrine-induced and calcium-induced cardiac hypertrophy in rats [57, 60–62]. Further studies are still required.

### 3.3. Histone Modification

Histone acetylation occurs at the lysine residues of the histone tails, resulting in the decondensation of the chromatin structure and acting as binding sites for bromodomain proteins and transcriptional activators, eventually leading to transcriptional activation [63]. A genome-wide analysis of histone markers that identify the epigenetic signatures of promoters and enhancers underlying cardiac hypertrophy indicated that the epigenetic landscape is a key determinant of the reprogramming of gene expression that occurs in cardiac hypertrophy [64]. The acetylation of histones is a dynamic process regulated by two enzyme families, the histone acetyltransferases and the histone deacetylases (HDACs). The balance between the activities of these two sets of enzymes is crucial for the regulation of gene expression, and its deregulation is linked to several pathological conditions in the development of HF. For example, sirtuins are a highly conserved family of histone/protein deacetylases and have been shown to participate in biological functions related to the development of heart failure, including regulation of energy production, oxidative stress, intracellular signaling, angiogenesis, autophagy, and cell death/survival. Emerging evidence indicates that two sirtuins (SIRT1 and SIRT3) play protective roles in failing hearts [65].

Thus, HDAC inhibitors have been suggested to restore the correct gene expression program in hypertrophied cardiac cells, as a prophylactic treatment for HF. Studies showed that cardiac fibrosis and hypertrophy were prevented by treatment with class I HDAC inhibitors [66–68]. HDAC inhibitors increase the acetylation of the sarcomeric proteins that enhance myofilament calcium sensitivity in cardiac cells [69]. Identifying the molecular targets of HDAC inhibitors could provide important information for the development of new drugs for cardiac hypertrophy and HF.

Surprisingly, another study revealed that estrogenic compounds derepressed the opposite effects of angiotensin II on the same parameters for HDAC4 and 5 (class II) [70]. This mechanism potentially supports the use of ER*β* agonists as HDAC modulators to treat cardiac disease.

The methylation of histones is a dynamic process mediated by histone methyltransferases and histone demethylases [71] and, unlike acetylation, histone methylation can either activate or repress gene expression, depending on the target site and the degree of methylation. The genome-wide histone methylation profile for HF showed that the trimethylation of histone H3 on lysine 4 (K4TM) or lysine 9 (K9TM) is markedly affected in cardiomyocytes during the development of HF in a rat disease model [72]. Another study concluded that HDAC4 plays an essential role in an acute increase of cardiac preload induced HDAC4 nuclear export, H3K9 demethylation, HP1 dissociation from the promoter region, and activation of the ANP gene and may represent a target for pharmacological interventions that prevent maladaptive remodeling in patients with HF [73].

### 3.4. MicroRNA-Based Mechanisms

MicroRNAs are single-stranded, about 22 nt-long ncRNAs that regulate gene expression mainly by forming partial hybrids with target mRNAs and thereby lowering their translation and/or stability [74]. A microRNA is transcribed as a long, primary miRNA (primiRNA) which is cleaved by the microprocessor complex to generate a miRNA precursor (premiRNA) that is exported to the cytoplasm [75]. In the last two decades, miRNA has fundamentally transformed our understanding of how gene networks are regulated and has become one of the most exciting areas in modern cardiological research. It was first discovered in 2007 that the increased expression of miR-21, miR-29b, miR-129, miR-210, miR-211, miR-212, and miR-423 and the reduced expression of miR-30, miR-182, and miR-526 are associated with human HF [76]. Since then, many miRNAs have been shown to be deregulated in specific tissues, playing critical roles in the pathogenesis and progression of HF. Four miRNAs are highly expressed in the heart: miR-1, miR-133, miR-208, and miR-499.

The main miRNAs involved are shown in Table 5. In studies by Ikeda et al. [77] and Sucharov et al. [78], the expression patterns of miRNAs in samples of myocardia from patients with ischemic cardiomyopathy, idiopathic cardiomyopathy, or aortic stenosis were analyzed. Interestingly, their results showed that subsets of miRNAs are differentially regulated in each of these etiologies.

Cardiac contractility depends on the expression of the two MHC isoforms, *α*- and *β*-MHC, and changes in their proportions may lead to hypertrophy, fibrosis, and the serious disruption of the contractile function of the heart. The increased expression of *β*-MHC in the myocardium, a common feature of cardiac hypertrophy and HF, may reduce the power output and can contribute to the depressed systolic function in end-stage HF [79]. Recently, an increase in *β*-MHC was associated with the overexpression of miR-208a in the heart, leading to arrhythmia, fibrosis, and hypertrophic growth in mice and poor clinical outcomes in humans with DCM [80]. miR-208a also controls systemic energy homeostasis by regulating the expression of MED13, suggesting a role for the heart in systemic metabolic control [81].

The downregulation of miR-1 is necessary to relieve the repression of growth-related target genes and induces hypertrophy [82]; miR-1 downregulates calcium-calmodulin signaling through calcineurin to nuclear factor of activated T-cells (NFAT) [83]. The reduction of miR-1 and increase in ANXA5 appear to be important modulators of NCX1 expression and activity during HF [84].

miR-195 is upregulated during hypertrophy. The cardiac overexpression of miR-195 in vivo can drive cardiac hypertrophy, which rapidly transitions to HF [85]. However, the mechanism by which miR-195 promotes hypertrophy is not well understood. Recent studies have shown that miR-195 potentially targets several genes involved in multiple signaling pathways, for example,* GADD45G*,* MAP2K1*,* MRAS*, and* RAF1*, which are involved in the MAPK signaling pathway [81]. Meanwhile, miR-195 targets the HMGA, MO25 gene, which is involved in apoptosis signaling [82, 83]. These findings suggest the potential mechanisms underlying the pathological role of miR-195 in hypertrophy.

miR-499 has been shown to enhance cardiomyogenesis in vitro and after infarction in vivo, which indicates that it enhances myocyte differentiation/hypertrophy [86]. Another study found that increased miR-499 in cardiac hypertrophy and cardiomyopathy is sufficient to cause murine HF and accelerates the maladaptation to pressure overloading in mice and humans [87]. These findings are similar to our finding that the expression of miR-499 increases after surgery for transverse aortic constriction. A bioinformatics analysis indicated that miR-499 might interfere with the WNT, JAK/STAT, and apoptosis signaling pathways during the development of hypertrophy [87, 88]. miR-23 and miR-24 were recently shown to be upregulated in hypertrophic and ischemic cardiomyopathy [77]. They show similar expression patterns and were predicted in this study to regulate MAPK and WNT signaling.

miR-21 is a miRNA that shows a consistent overexpression pattern in HF. The expression of miR-21 seems to be induced in endothelial cells by shear stress and regulates the function of vascular smooth muscle cells by modulating endothelial nitric oxide synthase (eNOS) activity [89]. Another study identified fibroblast exosomal-derived miR-21_3p (miR-21^∗^) as a potent paracrine-acting RNA molecule that induces cardiomyocyte hypertrophy. Proteome profiling identified sorbin, SH3 domain-containing protein 2 (SORBS2), and PDZ and LIM domain 5 (PDLIM5) as miR-21^∗^ targets and miR-21^∗^ silences SORBS2 or PDLIM5 expression in cardiomyocyte-induced hypertrophy [90]. Fibroblast-derived miR-21^∗^ is a paracrine signaling mediator of cardiomyocyte hypertrophy and a potential therapeutic target.

NFAT and miR-25 cooperate to reactivate the transcription factor HAND2 in HF [91]. Recently, Wahlquist et al. reported the pathological upregulation of miR-25 during HF and showed that its inhibition blocked and reversed the disease in mice. Although an increase in cardiac miR-25 levels caused a decline in cardiac function, antimiRNA-based inhibition of miR-25 halted established HF, at least in part, by increasing the mRNA of SERCA2a [92], suggesting that an inhibitor of miR-25 will be a potential therapeutic agent in the future. The above two studies show controversial data because of different chemistries and dose and at different times after the initiation of pressure-overload stress it is conceivable that miR-25 could play a beneficial role acutely by helping the heart adapt to pressure stress but produce long-term maladaptive effects. Future studies with expanded group sizes will be vitally important to further explore the therapeutic relevance of miR-25 inhibition in the setting of heart failure.

miR-133 is expressed in adult cardiomyocytes and skeletal muscle. Research showed that miR-133 levels reduced in the infarcted areas of the heart [93]. Among the miRNA altered in pressure-overload cardiac hypertrophy models, miR-133 was singularly downregulated [94]. Overexpressing miR-133 reduced apoptosis and increased viability of H9c2 cells after exposure to H_2_O_2_, whereas downregulating miR-133 expression with an inhibitory oligonucleotide promoted apoptosis in these cells and in neonatal rat ventricular cardiomyocytes [95]. Heart function has been restored by reprogramming nonmyocytes into cardiomyocytes, by expressing transcription factors (GATA4, HAND2, myocyte-specific enhancer factor 2C (MEF2C), and T-box 5 (TBX5)) and microRNAs (miR-1, miR-133, miR-208, and miR-499) [96], indicating that miR-133 could be a potential drug target for cardiac remodeling.

Expression of miR-199b was shown to be elevated in mouse models with pathological hypertrophy and in human failing hearts. da Costa Martins et al. recently described miR-199b involvement in an autoamplification loop promoting CN/NFAT signaling. Modulation of Dyrk1a by miR-199b constitutes a feed forward mechanism that enhances pathological cardiomyocyte hypertrophy processes [97]. Administration of antagomiR-199b to mice after transverse aortic constriction could reverse and/or attenuate pathological hypertrophy and fibrosis [97]. Further, we identified that the TWIST1/miR-199/214 axis is downregulated in dilated cardiomyopathy, which is likely to play a role in the increased activity of the UPS [98]. This may contribute to the loss of cardiac mass during dilatation of the heart. Besides, in vivo experiments using endothelial cell-specific MeCP2 null or Sirt1 transgenic mice confirmed the involvement of MeCP2/Sirt1 in the regulation of angiogenic functions of endothelial cells. TGF-*β* impairs endothelial angiogenic responses partly by downregulating miR-30a-3p and subsequent derepression of MeCP2-mediated epigenetic silencing of Sirt1 [99].

Recent evidence has shown that a proportion of circulating miRNAs are secreted from normal healthy or damaged cells as microvesicles. The fact that these circulating miRNAs can be detected in the peripheral blood makes them potentially useful in diagnosis or to guide therapy, with rapid and simple tests that eliminate the need for invasive procedures, such as biopsies [100].

Recent studies have shown that miR-103, miR-142-3p, miR-199a-3p, miR-23a, miR-27b, miR-324-5p, and miR-342-3p can be used to distinguish between HF, exacerbated chronic obstructive pulmonary disease, other causes of dyspnea, and controls [101]. The miRNAs miR-126 and miR-508-5p could also be useful in the diagnosis of chronic HF patients and might provide novel targets for the prevention and treatment of chronic HF [102]. FABP3, a miRNA target, can be used as an indicator of myocardial miRNA expression and function in human HF patients [103]. Other miRNAs that can be used as biomarkers for the diagnosis and prognosis of HF must be identified in future studies.

Currently, two therapeutic strategies involving miRNAs have been studied: the use of antimiRs and miRNA mimics (miR-mimics). In a pioneering study, Thum et al. found that an antimiR functionally designed to inhibit miR-21 significantly reversed the progression of cardiac hypertrophy and fibrosis and attenuated the impairment of cardiac function [104]. Another study by Montgomery et al. showed that the therapeutic inhibition of miR-208a prevented pathological myosin changes and cardiac remodeling, improving cardiac function and increasing survival [105]. The therapeutic efficacy of miR-mimics has also been studied. Suckau et al. successfully used a viral vector expressing optimized miR-mimics in mice to normalize cardiac dilation and significantly reduce cardiac hypertrophy and cardiac fibrosis [106]. Wahlquist et al. demonstrated that the increased expression of endogenous miR-25 contributes to the decline in cardiac function during HF and suggested that it might be targeted therapeutically to restore cardiac function [92]. More recently, Castaldi et al. found that miR-133 controls multiple components of the beta1AR transduction cascade and is cardioprotective during heart failure, which indicated overexpression of microRNAs in vivo is also a therapeutic strategy in the treatment of HF [107].

### 3.5. Long-Noncoding-RNA-Based Mechanisms

LncRNAs were discovered in the early 1990s and are nowadays defined as RNA molecules of >200 nucleotides in length [13]. LncRNAs regulate the expression of genes at the epigenetic, transcriptional, and posttranscriptional levels and play important roles in physiological processes. The fact that some lncRNAs have been found to be differentially regulated in the developing or diseased heart provides a strong indication for their involvement in cardiac (patho) physiology [13].

Wang et al. first demonstrated a novel cardiac-hypertrophy-regulating complex composed of the lncRNA CHRF, miR-489, and MYD88 [108]. Han et al. discovered that lncRNA protects the heart from hypertrophy through the BRG1-HDAC-PARP pathway and MHRT-BRG1 feedback. Similarly, the circulating lncRNA LIPCAR is a novel biomarker of cardiac remodeling and predicts the survival of patients with HF [109]. Mhrt is the first example, to our knowledge, of a lncRNA that inhibits myopathy and chromatin remodelers [109]. Moreover, transcription in the heart of Kcnq1 depends on the expression of the lncRNA Kcnq1ot1, which could be responsible for abnormal heart function [16]. ANRIL can also repress the expression of suppressor genes INK4b, ARF, and INK4a, which is involved in the development of coronary heart disease [15, 19, 110]. Also it is reported nowadays that the expression profiles of lncRNAs, but not mRNAs or miRNAs, can discriminate failing hearts of different pathologies and are markedly altered in response to LVAD support [111]. The mitochondrial long noncoding RNA, LIPCAR, has been proven to be a novel biomarker of cardiac remodeling and predicts future death in patients with heart failure [112]. CARL, a cardiac apoptosis-related lncRNA, can suppress mitochondrial fission and apoptosis by targeting miR-539 and PHB2, which may provide a new approach for tackling apoptosis and myocardial infarction [113].

Indeed, future studies on the role of lncRNA in HF and heart development will improve our understanding of the ncRNA network involved in regulating gene expression changes underlying HF and, thus, allow the development of specific therapeutic strategies based on the interference not only of miRNAs but also of lncRNA important for HF. These studies will greatly benefit from the combination of next-generation sequencing technologies applied to RNA (RNA-seq) with bioinformatic tools developed to identify lncRNAs that are differentially expressed in different biological conditions and for the redirection of their mechanism of action.

## 4. Conclusion

To understand the genetics and epigenetics of HF and their role in pathogenic cardiovascular processes is an exciting new frontier in cardiovascular medicine. Understanding the genetics of HF may not only allow its early detection but also make possible personalized medical care for HF. The dynamic aspects of epigenetics will provide more accurate evidence of the roles of changing environmental factors in drug responses, thereby linking the environment with the genome, and will also provide a way to reactivate silenced genes. The potential of miRNAs as new tools for diagnosis and prognosis is increasingly clear, and they offer promising therapeutic strategies for HF. Additional research is obviously required to clarify how epigenetic mechanisms affect the onset and development of heart disease and heart regeneration to identify new drug targets for HF and to allow disease classification and risk stratification.

## Figures and Tables

**Table 1:** Main dilated cardiomyopathy-causing genes dilated cardiomyopathy.

Gene	Protein	OMIM	% of familial DCM cases	Inheritancepattern
TTN	Titin	188840	15–27%	AD
LMNA	Lamin A/C	150330	6%	AD
MYH7	*β*-myosin heavy chain	160760	4.20%	AD
MYPN	Myopalladin	608517	3.50%	AD
TNNT2	Cardiac troponin T	191045	2.90%	AD

**Table 2:** Main dilated cardiomyopathy-causing genes hypertrophic cardiomyopathy.

Gene	Protein	OMIM	% of familial HCM cases
MYH7	Myosin heavy chain	160760	40%
	Cardiac muscle beta isoform	192600	

MYBPC3	Myosin-binding protein C, cardiac-type	600958	40%

TNNT2	Troponin T, cardiac muscle	115195	5%

TNNI3	Troponin I, cardiac muscle	191044	5%

TPM1	Tropomyosin 1 alpha chain	115196	2%
		191010	

MYL2	Myosin regulatory light chain 2	160781	Unknown
	ventricular/cardiac muscle isoform	608758	

MYL3	Myosin light polypeptide 3	160790	1%
		608751	

**Table 3:** Main arrhythmogenic right ventricular cardiomyopathy-causing genes hypertrophic cardiomyopathy.

Gene	Protein	OMIM	% of familial ARVC cases
DSP	Desmoplakin	125647	6%–16%
PKP2	Plakophilin 2	602861	11%–43%
DSG2	Desmoglein 2	125671	12%–40%

**Table 4:** Cardiomyopathy Genes and Associated Clinical Features.

Gene	DCM	HCM	ARVC	Inheritance	Location/role
Abcc9	X			AD	Potassium channel
ACTC1	X	X		AD	Sarcomere
ACTN2	X	X		AD	Z-disk
DES	X		X	AD	Intermediate filament
DSC2	X		X	AD	Desmosome
DSG2	X		X	AD	Desmosome
DSP	X		X	AD, AR	Desmosome
LAMP2	X	X		XL	Lysosome
LMNA			X	AD	Nuclear membrane
MYBPC3	X	X		AD	Sarcomere
MYH7	X	X	X	AD	Sarcomere
PKP2		X	X	AD	Desmosome
TNNC1	X	X		AD	Sarcomere
TNNI3	X	X		AD	Sarcomere
TNNT2	X	X		AD	Sarcomere
TTN	X	X	X	AD	Sarcomere

**Table 5:** Main involved miRNAs in heart failure.

MicroRNA	Expression in HF	Function in cardiac vascular system
1	Downregulated	Development and function of cardiac and skeletal muscle
15/16	Upregulated	Apoptosis induction
21	Upregulated	Induced in endothelial cells by shear stress; modulates the apoptosis and eNOS activity
195	Upregulated	Involved in myocyte hypertrophy and dilated cardiomyopathy
199a	Upregulated	Essential for maintaining the cardiomyocytes size
133	Downregulated	Development and function of cardiac and skeletal muscle; Regulation of beta-adrenergic receptors
23a	Upregulated	Involved in the regulation of cardiac hypertrophy
320	Upregulated	Involved in the regulation of cardiac ischemia injury
208	Upregulated	Stress-induced cardiac hypertrophy, Reduced *β*-MHC expression
